# Self-medication and Anti-malarial Drug Resistance in the Democratic Republic of the Congo (DRC): A silent threat

**DOI:** 10.1186/s41182-022-00466-9

**Published:** 2022-10-04

**Authors:** Aymar Akilimali, Charles Bisimwa, Abdullahi Tunde Aborode, Chrispin Biamba, Leonard Sironge, Alain Balume, Rahma Sayadi, Samuel Babatunde Ajibade, Akintola Ashraf Akintayo, Tolulope Olamide Oluwadairo, Emmanuel Adebowale Fajemisin

**Affiliations:** 1 Faculty of Medicine, Official University of Bukavu, Bukavu, DR Congo; 2 Faculty of Pharmacy and Public Health, Official University of Bukavu, Bukavu, DR Congo; 3 Healthy Africans Platform, Research and Development, Ibadan, Nigeria; 4 Faculty of Medicine, University of Goma, Goma, DR Congo; 5 Faculty of Medicine, University of Monastir, Monastir, Tunisia; 6 Department of Environmental Science, Tennessee State University, Nashville, TN USA; 7 Department of Biomedical Convergence Science and Technology, Kyungpook National University, Daegu, 41566 South Korea; 8 Texas A&M University-Kingsville, Texas, USA; 9 Department of Integrative Biomedical Sciences, University of Cape Town, Cape Town, South Africa

**Keywords:** Antimalarials, Drug resistance, Malaria, Self-medication, Democratic Republic of Congo

## Abstract

**Background:**

Malaria is a global infectious (vector-borne: Anopheles mosquitoes) disease which is a leading cause of morbidity and mortality in Sub-Saharan Africa (SSA). Among all its parasitic (protozoan: *Plasmodium sp.*) variants, *Plasmodium falciparum (PF)* is the most virulent and responsible for above 90% of global malaria deaths hence making it a global public health threat.

**Main context:**

Despite current front-line antimalarial treatments options especially allopathic medications and malaria prevention (and control) strategies especially governmental policies and community malaria intervention programs in SSA, *PF* infections remains prevalent due to increased antimicrobial/antimalarial drug resistance caused by several factors especially genetic mutations and auto(self)-medication practices in SSA. In this article, we focused on the Democratic Republic of Congo (DRC) as the largest SSA country by bringing perspective into the impact of self-medication and antimalarial drug resistance, and provided recommendation for long-term improvement and future analysis in malaria prevention and control in SSA.

**Conclusions:**

Self-medication and anti-malarial drug resistance is a major challenge to malaria control in DRC and sub-Saharan Africa**,** and to achieve sustainable control, individual, community and governmental efforts must be aligned to stop self-medication, and strengthen the health systems against malaria.

## Introduction

Malaria has been a scourge of humanity since antiquity and remains with an annual morbidity–mortality of approximately 229 million cases and more than 400,000 deaths [1, 2]. For the global fight against malaria, the beginning of this century has been marked by a series of steady advances that have averted millions of cases and saved millions of lives [3]. In 2020, no progress was made in this fight [1]. This could be linked to the spread of Plasmodium strains resistant to anti-malarial drugs and access to good quality drugs [4]. One in ten Antimalarial medications in developing countries is either of poor quality or falsified [3].

Nevertheless, *Plasmodium falciparum* parasites have developed resistance to all effective anti-malarial drugs, including Chloroquine (CQ), Sulfodoxine–pyriméthamine (SP), and Artemisinin with its derivatives [5]. Over the years, quinine and artemisinin are the two basic classes of antimalarials in antimalarial chemotherapy whose curative and prophylactic efficacy serves as golden standards, but current treatment involves combinational therapies especially artemisinin-based combination therapies (ACTs) [6]. Aside genetic mutations, self-medication with antimalarials remains one of the main factors in developing resistance to antimalarials within the population living in endemic areas [7].

This review article focuses on self-medication and antimalarial drug resistance in malaria cases caused mostly by *Plasmodium falciparum (PF)*in the Democratic Republic of Congo (DRC), and provide recommendations that may be important to the Congolese government and the Congolese population and can be gradually adapted to fight against self-medication of anti-malarial drugs, to limit the resistance from the wrong use of Antimalarials in DRC.

## Methods

Specific keywords (anti-malarial drugs, effects, self-medication, democratic republic of Congo, resistance) were subjected to list down and analyzed the literature from Pubmed–Medline and Google Scholar. Given the commentary character of the present report, all types of peer-reviewed records, including original research, systematic reviews, and meta-analysis, were considered. The focus was given on studies published between the years 2003–2022. However, older studies with findings elucidating the resistance of anti-malarials sequelae were also included where appropriate. We excluded outdated studies and studies reporting data and scholarly research that is not in line with the objective of the study and the keywords of the search. The reference lists of the included studies were also hand-searched to identify additional relevant records. Out of all articles, as per inclusion criteria, we selected 40 papers for further analysis and narrative to build on this research study.

### Epidemiology of Malaria in DRC

According to the 2020 World Health Organization reports, malaria was estimated to 241 million cases globally and resulted in 627,000 deaths [8]. In the same year, Africa accounts for a large and disproportionate part of the global malaria burden with 95% of malaria cases and 96% of deaths from the disease have been recorded, of which children under 5 are victims and account for 80% of all deaths [8].

Roughly 30 million cases of this curable and avoidable disease affect nearly 95% of the population or about 69 million individuals who live in malaria-endemic areas [23]. Of the 620,000 children in the DRC who do not reach their fifth birthday, over half die from malaria [23].

The emergence and spread of antimalarial drug resistance are posing tremendous challenges for malaria treatment management. Before 2003, chloroquine was used as a first-line antimalarial treatment in DRC due to its cost-effectiveness; however, a 2003 study by Walters and colleagues compared the efficacy of chloroquine to its available alternative, sulfadoxine–pyrimethamine (SP) in uncomplicated plasmodium falciparum malaria in DRC [30]. The study framework aligned to standard WHO 14-day in vivo test reported an unacceptable CQ treatment failure rate and excellent efficacy of SP, hence confirming chloroquine resistance in DRC, and SP as an alternative treatment [25, 26, 30]. Over the years, SP and other emerging antimalarials have lost their efficacy against Plasmodium falciparum. Indeed, many factors contribute to the development and spread of resistance, especially gene mutations (parasite) and drug pressure (self-medications from users) [27].

Approximately 11% of the world's malaria load is found in the Democratic Republic of the Congo (DRC) [24]. DRC established a nationwide initiative called National Malaria Control Program (NMCP) to combat malaria in 1998 [25]. The primary goal of this initiative was to decrease malaria-related deaths by 50% by 2015 and malaria-related morbidity by 25%. Insecticide-treated net (ITN) distribution, indoor residual spraying promotion, intermittent prenatal care promotion and implementation, rapid diagnostic test promotion, and community and mother case management with artemisinin-based combination therapies are all examples of interventions included in the NMCP's activities [25, 26].

The NMCP has increased their efforts to combat malaria over the past 15 years [27]. From 271 out of 516 health zones in 2009, the NMCP now covers all 516 health zones across the country in 2016 [27]. In addition, in 2003 the NMCP set up a network of 11 sentinel locations for integrated surveillance of priority diseases, such as malaria; by 2016, this network had expanded to 26 new provinces [28].

The democratic republic of Congo represents one-tenth of the burden of malaria in Africa, with more than 15 million cases and 25,000 deaths recorded each year [9]; this represents 12% of malaria cases in Sub-Saharan Africa [9]. As a result, in DRC, malaria remains the leading cause of morbidity and mortality, accounting for more than 40% of all outpatient visits and 19% of deaths among children under five [8, 9]. This situation of high malaria burden is explained by the fact that almost the entire population lives in areas of high transmission, where the vector *Plasmodium falciparum* is the most common species responsible for the majority of severe cases [10].

### Effects of Self-medication on Anti-malarial drug in DRC

According to a study conducted on 515 people residing in one of the major cities of the DRC, Lubumbashi, self-medication has a prevalence of 99%, with 82.4% of subjects who practice self-medication in the event of malaria, and 79.4% use quinine as the drug for self-medication. In this region, a heightened antimalarial multi drug resistance was observed in patients and drug dependence/addiction (CQ/SP) were reported [11]. Throughout the DRC, the frequency of self-medication is exceptionally high due to the free sale of quinine and Artemisinin derivatives in pharmacies beyond the reliability standards that the ministry of public health offers [12].

Several factors related to self-medication can be observed between the lack of means to pay for drugs in pharmacies that meet government standards and the lack of means to access the health care service. For lack of means, some resort to traditional medicine in the event of malaria using an infusion or a decoction based on the bark of Quinquina *(Cinchona officinalis).* Plasmodium falciparum develops resistance to several drugs globally, so its resistance only targets quinine and Artemisinin derivatives (the most widely used antimalarials in our region) [13].

Studies indicated that self-medication is associated with inappropriate treatments and polychemotherapy [12, 13]). In Bukavu, a town in the eastern region of the DRC, and elsewhere in the country, there are no regulations on the sale and use of pharmaceuticals, which then results in a high incidence of self-medication of Antimalarial drugs [11].

Wasting drug availability, pathogen resistance (which can lead to serious health dangers, such as adverse responses), additional suffering, and drug dependence are all issues that arise from people trying to treat themselves [17]. The widespread availability of antimalarial drugs for self-prescription is a direct result of the lack of oversight in the pharmaceutical industry [17]. There is a possibility that this could lead to overuse and the development of resistant pathogens [17, 18].

The rise of antimalarial-resistant parasite as a global health crisis has left doctors in every part of the world grappling with difficult therapeutic questions. This issue has become a global crisis due to the general public's lack of knowledge on the topic and the ineffectiveness of existing alternatives to anti-malarial drugs [19]. Inappropriate use of antimalarial medications can cause a chain reaction in *Plasmodium* parasite*,* leading to an ecological phenomenon known as antimalarial drug resistance [20].

Human pathogens frequently acquire resistance to Antimalarial medications due to self-medication [21]. Inappropriate or incorrect treatment, or even missed diagnosis, can lead to bacterial resistance problems and high mortality, as can the widespread use of unnecessary antimalarial drugs and a lack of knowledge about Antimalarial courses, side effects, an approach which provides potency boundaries, and antibiotic overdose concerns [21]. Skin issues, severe allergies, hypersensitivity, etc., are only some of the numerous potential side effects of an overdose on anti-malarial medication [22]. There could be fewer cases of microbial resistance around the world if the general public had a better grasp of allopathic medications, particularly anti-malarials [22].

### Fight against malaria and Anti-malarial drug Resistance 

At the global level, different programs have been created to support malaria elimination in certain countries [12]. It is a high-level plan to provide lifesaving interventions to all populations at risk of malaria and reduce the spread of resistant parasites [3, 11].

The abuse of antimalarial self-medication has caused people to defer hospital intervention, which has further complicated their illnesses. Self-medication may relieve the problem but not the disease condition, as some diseases, such as typhoid fever, have common characteristics with malaria [15]. Self-medication is prevalent for a variety of reasons, including cultural norms, financial constraints, a lack of information about the effects of self-medication, personal knowledge of the existing signs, the expense of hospital visits, a busy schedule, a lack of transportation, the location of the nearest health care facility, the severity of the illness, the immediacy of the situation, red tape, crowding, a lack of medication supplies, and the nature of the illness itself [16].

In the DRC, 14 million mosquito nets impregnated with long-lasting insecticide were distributed in ten of the country's provinces during awareness campaigns [4, 8]. Nearly 2.5 million pregnant women benefit from two doses of Fansidar (an uncomplicated *Plasmodium falciparum* antimalarial), and almost 2 million others have benefited up to the third dose [9], and these women benefit from this care during the prenatal consultation [9, 12, 13]. In a report, 20% of students were examined after self-medication with antimalarials [35]. The high figure shows the existence of a gap in the health care system including the freedom to buy drugs without a prescription in pharmacies [35].

When treating uncomplicated *P. falciparum* malaria, the World Health Organization (WHO) suggests checking in on the drug's effectiveness every 2 years [29]. If a drug's effectiveness drops below 10%, the WHO recommends switching to a different treatment [29]. Chloroquine (CQ) has been phased out for the treatment of uncomplicated P. falciparum malaria in endemic regions because of its diminishing therapeutic efficiency. A clinical trial conducted in 2001 in the DRC to assess the efficacy of CQ found a treatment failure rate of 45.5% [30]. This high failure rate led to the replacement of CQ by the combination sulfadoxine–pyrimethamine (SP) in the management of uncomplicated malaria [31]. In 2005, due to the declining efficacy of the present medicines, the DRC adopted the use of artemisinin-based combination therapy (ACT) as the first-line treatment for uncomplicated malaria [31]. Artesunate–amodiaquine (ASAQ) has been the mainstay of the country’s ACT anti-malarial medication policy since the inception of ACT in the DRC. It was later in 2012 when artemether–lumefantrine (AL) was introduced as an alternative to ASAQ [32]. Uncomplicated *P. falciparum* malaria in the DRC is being treated using a combination of ASAQ and AL [32]. However, based on molecular studies, clinical antimalarial resistance to antimalarial medications has also been linked to genetic mutations, and these published studies had revolved around five [5] major mutated genes [36]: Chloroquine (CQ) and Amodiaquine (AMQ) resistance was linked to mutants of *Plasmodium falciparum* chloroquine resistant reporters (*pfcrts*) genes which efflux chloroquine from the digestive vacuole (drug action site), and further strengthen parasite fitness including altering their susceptibility to front-line malaria therapies [33, 34, 36]; *Plasmodium falciparum* dihydropteroate synthase gene (pfdhps) was linked to sulfadoxine resistance; *Plasmodium falciparum* dihydrofolate reductase genes (pfdhfr) was linked to pyrimethamine; *Plasmodium falciparum* multidrug resistance 1 (pfmdr1) gene was linked to many drugs including lumefantrine (LMF), mefloquine (MQ), AMQ, and even artemisinin resistance; and kelch-13 (K-13) propeller gene linked to artemisinin and artemisinin-based combination therapy (ACTs) resistance [36].

## Conclusions and future recommendations

With the high frequency of cases of morbidity and death due to malaria, the Congolese population is threatened because of the problems of self-medication and the resistance to antimalarials. For the Congolese government, we recommend cleaning up the pharmaceutical sector to curb the sale of products of dubious quality (damaged, counterfeit, or falsified), ensure the management and conscious use of the antimalarial arsenal at the fundamental level of pyramid care, allocate a budget to support research into antimalarials, encouraging innovation in natural molecules. It is needed to provide mosquito nets infused with long-lasting insecticides to prevent new malaria cases in the country [14, 35].

It is significant to carry out permanent training of health workers on the proper use of antimalarials and, in particular, the owners of pharmacies in the materialization of the fight against self-medication, a critical factor in the emergence of resistance to antimalarials and adopt and enforce regulations restricting the over-the-counter sale of systemic and broad-spectrum antimalarials without a prescription from a qualified and recognized healthcare provider.

The health system in the DRC must organize awareness campaigns among the population on the imminent danger represented by diseases resistant to anti-malarials drugs. At the same time, it will discourage the practice of self-medication and offering quality health care by not taking into account the socio-economic characteristics of the patients. The Congolese population must respect the regulations enacted by the political and health authorities concerning prevention, the fight against resistance to antimalarials, and the ban of anti-malarials drugs without Medical professionals' recommendation. It is important to carry out frequent sanitation in the environment, where it is due for mosquito control.

## Data Availability

Not applicable.
